# Cottonseed oil composition and its application to skin health and personal care

**DOI:** 10.3389/fphar.2025.1559139

**Published:** 2025-03-19

**Authors:** Janelle Gutierrez, Slavko Komarnytsky

**Affiliations:** ^1^ Plants for Human Health Institute, NC State University, Kannapolis, NC, United States; ^2^ Department of Food, Bioprocessing, and Nutrition Sciences, NC State University, Raleigh, NC, United States

**Keywords:** fatty acid profile, carrier oil, skin barrier function, skin conditioning, sebum, emulsions, formulations, cosmetic applications

## Abstract

The historical use of oils for beauty and hygiene dates back to ancient civilizations. While mineral oil and its derivatives dominated the personal care industry in the 20th century due to chemical stability and low cost, the environmental impact and sustainability concerns have driven a resurgence in the use of vegetable oils. Cottonseed oil derived from *Gossypium hirsutum* L. (*Malvaceae)* has been often overlooked in favor of other plant oils, likely due to cotton’s primary use as a fiber crop. Yet cottonseed oil stands out in cosmetics for its beneficial linoleic to oleic acid ratio, which supports skin barrier function, and its rich profile of phytosterols and tocopherols that provide higher oxidative stability and extended shelf life. Cottonseed oil is also adaptable for use in a variety of formulations, offering a lightweight, non-greasy emollient base with potential applications in skin care, hair, and cleansing products. This review highlights cottonseed oil as a potentially underutilized ingredient in the personal care sector and emphasizes the need for further research and development to fully exploit its properties.

## 1 Introduction

Oil is a common ingredient found in many cosmetic and personal care products. In the past, oils were extracted from natural sources including animal and vegetable fats based on their geographical distribution, accessibility, and changes in contemporary agricultural practices associated with animal and crop domestication (Komarnytsky et al., 2022). While these oils may not have been processed on a large scale like in later periods, they still played an essential role in personal care and practices (Moore et al., 2020). Among the earliest records of using natural oils for skin care, and in balms and ointments by the Mesopotamian and Mediterranean cultures, are olive (*Olea europaea* L.), sesame (*Sesamum indicum* L.), castor (*Ricinus communis* L.), laurel (*Laurus nobilis* L.), and argan (*Argania spinosa* L.) oils. The lesser-known oils of pre-Columbian Americas include avocado (*Persea americana* Mill.), sunflower (*Helianthus annuus* L.), cotton (*Gossypium hirsutum* L.), as well as jojoba (*Simmondsia chinensis* (Link) C. K.) liquid wax esters.

This trend has changed abruptly when mineral oil was discovered as a byproduct of refined crude oil in the mid-19th century. Its low cost, chemical stability and inertness rapidly established it as an ideal base ingredient in skincare formulations. Refined mineral oils are colorless, odorless, and tasteless lipids that have been safely used in cosmetics for many decades to achieve occlusive moisturizer (non-absorbent) and emollient (skin softening) outcomes (Rawlings and Lombard, 2012). Common ingredients that are mineral oil-based are petrolatum, paraffin, microcrystalline cera and wax, ceresin, and ozokerite. While these promote healthier skin, their major downsides such as environmental contamination of water and soil, lack of biodegradability, and limited sustainability revitalized the search for alternative ingredients that have similar skin benefits (Juliano and Magrini, 2017). In the 21st century, consumers demanded better, longer-lasting, and safer personal care products that are cruelty-free and environmentally friendly (Amberg and Fogarassy, 2019). In response, the industry has once again increased the use of vegetable and seed oils in their formulations (Bom et al., 2019).

Allotetraploid *Gossypium hirsutum* L. (tribe *Gossypiae*, family *Malvaceae*), commonly known as upland cotton native to Mesoamerica, emerged within the last 1–2 million years, resulting from the unlikely transoceanic dispersal of an A-genome species to the New World, where it hybridized with a native D-genome diploid (Wendel and Grover, 2015). It was brought into cultivation as early as 3,500 BC in the Tehuacan Valley of Mexico (Smith and Stephens, 1971), and further developed into a vigorous cotton industry in the lowland Veracruz by 300 AD (Stark et al., 1998). Botanical remains of cotton seeds and embryos were found in three huts at the Ceren site in El Salvador, which was buried by volcanic ash from the nearby Loma Caldero volcano (585-600 AD). These remains, especially those found on a metate surface in hut 4, strongly suggest that the Ceren inhabitants were grinding cotton seeds to extract oil (Lentz et al., 1996). Cottonseed DNA was also detected in the Huecoid coprolite sample from Vieques, Puerto Rico dated to 500 AD, although it is not clear whether it was consumed or introduced via saliva during spinning of cotton fibers (Reynoso-García et al., 2023). A more recent use of the crushed cotton seeds (*taman*) by Yucatecan Maya to manage tenesmus may have provided an alternative medicinal explanation to these findings (Roys, 1931). Scalp diseases, ulcers, and other skin conditions were also routinely treated with boiled and crushed cotton leaves and flowers in this region (Roys, 1931). Elsewhere, Pima Indians from American Southwest consumed ground or parched cotton (*taki*) seeds (Russell, 1908). Although cotton could only be cultivated in the limited ecological zones defined by a narrow range of warm temperatures (11°C–25°C) and a rainy period followed by dry weather, extensive trade networks ensured that cotton (*ichcatl*) was accessible to elites across most regions of the Americas during the Aztec empire in the early 16th century (Berdan, 1987). Other species of cotton were developed in South Asia (*Gossypium arboreum* L.) and Northeast Africa (*Gossypium herbaceum* L.) (Fuller et al., 2024), but historical records of cottonseed oil use in these regions prior to modern times are virtually unknown, much like with soybeans.

Cotton seeds contain a significant amount of oil with a high-quality fatty acid composition (Riaz et al., 2023a). Rich in linoleic, palmitic, and oleic fatty acid, sterols, tocopherols, and other beneficial compounds, cottonseed oil has moisturizing, antioxidant, and anti-inflammatory properties that can benefit personal care (Egbuta et al., 2017). Historically, cottonseed oil has not received the same recognition as other plant-based oils, likely due to cotton’s primary role as a fiber crop rather than an oilseed, and its potential in the cosmetic industry remains largely untapped. Cottonseed oil is a readily available, inexpensive agricultural byproduct that is regulated under strict food-grade standards, making it an eco-friendly alternative to more expensive oils. In this review, we aim to highlight the underutilized potential of cottonseed oil in skin and personal care product development, and accentuate the opportunities it presents as a sustainable, eco-friendly ingredient. The analysis emphasizes distinctive balance of linoleic and oleic acids, oxidative stability, and formulation versatility of cottonseed oil, shifting the focus from its traditional role in agriculture to its overlooked cosmetic potential.

## 2 Cottonseed oil extraction and refining

**FIGURE 1:**
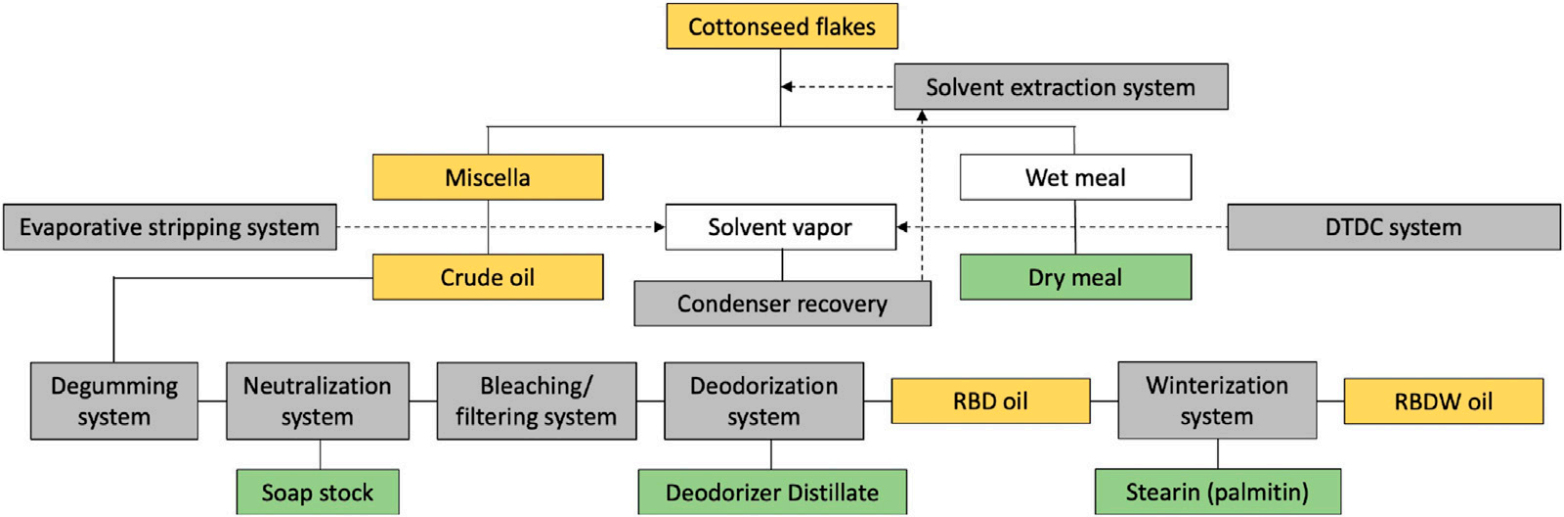
Cottonseed oil manufacturing flowchart. Oil-containing materials are highlighted in yellow as crude, RBD (refined, bleached, deodorized), and RBDW (RBD winterized) oils. The byproducts remaining after the processing steps are highlighted in green.

Whole cottonseed on average contains about 16% crude oil, 45% meals, 27% hulls, 8% linters, and 4% waste, while its dehulled meat (kernel) contains about 37% crude oil and 41% protein (He et al., 2023). The cottonseed oil available on the market is mostly refined; both solvent and mechanically expressed crude oils can be used in the refining process (Riaz et al., 2023b). Refining also accomplishes the removal of a naturally present terpenoid aldehyde gossypol and cyclopropenoids such as sterculic (CPE 19:1) and malvalic (CPE 18:1) fatty acids. Both components are considered undesirable for nutritional and health purposes (Yang et al., 2021). This is similar to rapeseed oil that was naturally high in erucic acid until it was cross bred to produce canola cultivars with a very low erucic acid trait (Gu et al., 2008). The refined cottonseed oil also contains up to 2% of a variety of nonoil substances (Andersen, 2001), while several volatile substances end up in the deodorizer distillate byproduct (Hussain Sherazi et al., 2016) (Figure 1).

Refined cottonseed oil can be further modified through winterization, a fractional crystallization process that slowly cools oil and removes the precipitating higher melting stearin (palmitin) byproduct. Winterization produces oil that remains clear at lower temperatures, with a pleasant flavor and increased stability (Khan et al., 2021a). The winterization process is very successful in cottonseed oil because it contains only trace amounts of the linolenic acid in contrast to canola and soybean oils. The cottonseed oil can be also modified through full hydrogenation or interesterification to harden it without generating trans-fatty acids (Imran and Nadeem, 2015). Multiple types of the cottonseed oils are therefore available to the manufacturers and consumers to tailor the physiochemical and functional properties of their formulations (Table 1).

**TABLE 1:** General differences among vegetable oils based on extraction and refining methods applied.

Cold-pressed^a^ ^,^ ^b^	Virgin^a^ ^,^ ^b^	Crude (whole)^a^	Refined^a^	Winterized^a^	Hydrogenated^a^
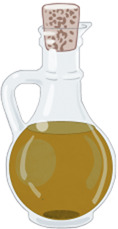	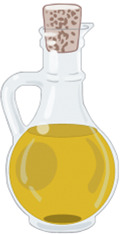	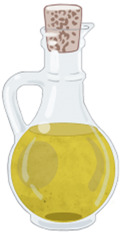	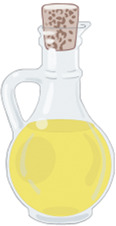	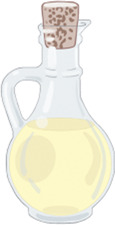	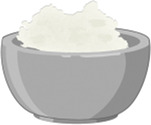
Mechanical below 30°C	Mechanical with heat	Solvent extraction	All oils can be refined	Chilled	Processed
High flavor, phytochemicals	Moderate	Moderate	Neutral	Neutral	Solid at RT
High compositional variability	Moderate	Low	Uniform	No stearin	No M/PUFAs
Most susceptible to oxidation	More susceptible	Less susceptible	High stability	Improved	Highly stable

^a^ All vegetable oils can be produced at a pharmaceutical (USP, NF), food (FCC), cosmetic, feed (AAFCO), or technical grades.

^b^ Cottonseed oil is typically not manufactured as a cold-pressed or virgin oil on a large scale.

Both environmental conditions and agronomic practices, such as temperature, rainfall, or nitrogen and phosphorus availability can alter lipid metabolism and modulate oil content of the seeds (Miklaszewska et al., 2021). *De novo* fatty acid synthesis is catalyzed by a complex of several enzymes in plastids, where metabolic flux redirections directly affect fatty oil composition (Song et al., 2022).

Different cotton cultivars and species vary in oil content and fatty acid profile. Cotton varieties with an ultra-low seed gossypol trait have been recently approved for human use by the FDA (Rathore et al., 2020), and there are some early indications that breeding low cyclopropenoid fatty acid cotton without negatively influencing other compositional factors of cottonseeds may be possible (Dowd et al., 2010a). When successful, these developments hold the potential to establish additional use of cold-pressed or virgin cottonseed oils in cosmetic formulations. Cold-pressing of cottonseed oil can be also facilitated with multi-enzyme applications (Latif et al., 2007).

## 3 Cottonseed oil components that support skin health

**FIGURE 2:**
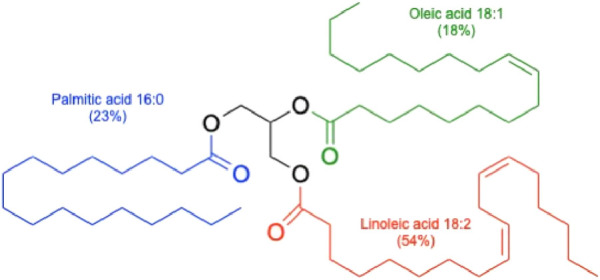
Schematic triacylgliceride of cottonseed oil. Different ratios of fatty acid esters are indicated for the three major fatty acids found in cottonseed oil.

The cottonseed oil is classified as a liquid seed oil similar to avocado, canola, soybean, hemp, and sunflower oils and in contrast to palm, coconut, and cacao bean oils that remain solid under the normal storage conditions. The cottonseed oil is also a semi-drying oil, similar to canola and unlike non-drying (olive, almond) or drying botanical oils (soybean, hemp, sunflower). Semi-drying oils slowly react with oxygen in the air to form soft elastic films in contrast to dying oils that harden to solid rigid films, and non-drying oils that remain as sticky liquids (Orlova et al., 2021). All vegetable oils are available as a natural mix of triglycerides with different ratios of fatty acid esters, and the latter define the bulk of their physicochemical properties (Figure 2).

### 3.1 Fatty acids

Cottonseed oil has a different fatty acid composition compared to other vegetable oils widely used in cosmetic and personal care formulations. This oil is dominated by the linoleic acid similarly to the spectrum of high-linoleic vegetable oils that include sunflower, hemp, soybean, and rosehip oils, among others. However, cottonseed oil harbors a saturated palmitic acid as the second most abundant fatty acid ester. This is a rather uncommon feature among the linoleic acid-dominated vegetable oils, and a similar high amount of palmitic acid can be found only in avocado, which is a high-oleic oil (Table 2). Both naturally-occurring (Shockey et al., 2017) and transgenic (Chapman et al., 2001) high oleic acid cotton cultivars have been also reported, but not grown widely.

**TABLE 2:** Cottonseed oil among the selected vegetable oils based on their major fatty acid profiles (%).

Botanical source	Almond kernel	Olive drupe	Rapeseed seed	Avocado fruit	Argan kernel	Roshi fruit	Soybean seed	Cotton seed	Hemp seed	Sunflower seed
	*Prunus dulcis*	*Olea europaea*	*Brassica napus*	*Persea gratissima*	*Argania spinosa*	*Rosa canina*	*Glycine max*	*Gossypium hirsutum*	*Cannabis sativa*	*Helianthus annuus*
Fatty acid profile^1^, %										
Palmitic acid 16:0	4–9	8–20	3–6	5–25	10–15	2–5	7–14	17–28	4–13	4–9
Palmitoleic acid 16:1 n-7	1	3	trace	1–13	trace	trace	trace	1–2	1	1
Stearic acid 18:0	2–3	1–5	2–3	2–3	4–7	2–3	1–6	1–4	1–4	1–7
Oleic acid 18:1 n-9	62–86	56–85	50–67	50–74	43–50	13–18	17–30	13–25	6–21	14–40
Linoleic acid 18:2 n-6	7–30	4–20	16–30	6–20	29–37	35–50	44–62	45–63	46–66	48–74
α-Linolenic acid 18:3 n-3	1	1	6–14	3	trace	22–38	4–11	trace	14–30	trace
γ- Linolenic acid 18:3 n-3	0	0	0	0	0	0	0	0	3–4	0
Saturation ratio^b^	8:71:21	17:69:14	6:61:32	22:66:12	19:46:35	6:15:79	18:24:58	26:18:54	8:12:82	11:16:71
Physicochemical properties										
Color	Yell	Yell Grn	Yell Brn	Drk Grn	Yell Orn	Orn Red	Yell Brn	Lt Yell	Drk Grn	Drk Yell
Refractive index	1.469	1.469	1.473	1.469	1.471	1.480	1.475	1.473	1.479	1.474
Density, g/cm^c^	0.915	0.913	0.917	0.916	0.915	0.920–950	0.922	0.918	0.926	0.921
Melting point, °C	−18	−6	−10	−2	1	−24	−17	−1	−8	−11
Iodine value^c^, g/100 g	90–100	75–88	94–106	75–95	91–105	180–190	128–135	103–111	150–167	118–144
SAP value^d^, mg/g	188	190	174	186	191	187	191	194	193	189
Unsaponifiable frac., %	0.9	1.5	1.5	2.0	1.5	1.5	1.5	2.0	1.5	1.5
Absorption speed	3	3	2	4	3	1	3	3	3	3
Comedogenic value	2	2	1	3	0	1	5	3	0	1
Soapmaking qualities										
Hardness	7	17	6	22	15	6	16	26	8	11
Conditioning	89	82	91	70	81	89	82	71	90	87
Cleansing	0	0	0	2	1	0	0	0	0	0
Creaminess	7	17	6	22	14	6	16	26	8	11
Bubbliness	0	0	0	0	1	0	0	0	0	0
INS^e^	97	105	56	99	95	10	61	89	39	63

^a^ Fatty acid composition (influences skin feel and absorption).

^b^ Ratio of saturated (SFA), monounsaturated (MUFA), and polyunsaturated (PUFA) fatty acid esters.

^c^ Amount of iodine (g) absorbed by 100 g of oil (the degree of unsaturation, affecting stability and drying time).

^d^ Amount of potassium hydroxide (mg) required to saponify 1 g of oil (the average molecular weight of fatty acids).

^e^ Calculated number derived by subtracting the iodine value from SAP (hardness, lather, and conditioning).

Several physiochemical characteristics collectively influence the oil’s texture, skin feel, penetration ability, and stability in cosmetic formulations. Most vegetable oils are produced with an acid value of 2–4 mg KOH/g and a peroxide value of 10–20 meq O_2_/kg. These parameters indicate free fatty acid content and oxidative rancidity, serving as markers for oil freshness, stability, and shelf life. Refined cottonseed oil typically exhibits improved quality parameters (acid value: 0.5 mg KOH/g; peroxide value: 5–10 meq O_2_/kg), resulting in higher oxidative stability and an extended shelf life when used in formulations (Taghvaei et al., 2014).

Another important property influenced by the composition of major fatty acid esters is the iodine value, which serves as a key indicator of hydrothermal and oxidative stability. Higher iodine values indicate greater unsaturation and lower stability. Cottonseed oil has the lowest iodine value among high linoleic acid oils, with values only slightly higher than those of almond, canola, and argan oils (Table 2), which is particularly beneficial in formulations where stability is crucial.

#### 3.1.1 Linoleic acid 18:2 (omega-6)

Linoleic acid is an essential fatty acid in the full-thickness human epidermis, making up 22%, followed by oleic acid (15%), palmitic acid (14%), and stearic acid (11%). However, this composition shifts in sebaceous secretions, which contain only trace amounts of linoleic acid but are rich in palmitic acid (22%), sapienic acid (22%), oleic acid (15%), and myristic acid (13%), as summarized previously (Knox and O’Boyle, 2021).

The linoleic acid is selectively utilized by sebaceous cells for β-oxidation, serving as a unique energy source for their function (Pappas et al., 2002). Additionally, linoleic acid metabolites are released following skin exposure to chemical irritants, suggesting the presence of a natural skin defense mechanism based on linoleic acid (van de Sandt et al., 1995). Within cellular metabolism, linoleic acid acts as a precursor to signaling molecules like arachidonic acid, which can regulate inflammatory responses (Komarnytsky et al., 2021). Lower skin linoleic levels are also associated with psoriasis (Mysliwiec et al., 2019) and acne (Zhou et al., 2018) skin pathologies.

#### 3.1.2 Palmitic acid 16:0 (saturated)

Similar to linoleic acid being an important link to cell lipid metabolism that helps maintain skin barrier integrity and resilience, exogenously supplied palmitic acid can be desaturated at an unusual C6 position to produce sapienic acid 16:1 (n-10) and undergo elongation to sebaleic acid 18:2 (n-10) in human sebaceous glands (Ge et al., 2003). The synthesis of sapienic acid and wax esters, along with the accumulation of squalene and the presence of very long-chain branched or hydroxylated fatty acids, are unique to sebum and rarely occur in other organs (Pappas, 2009). Due to its occlusive nature, palmitic acid is effective in sealing moisture, which is advantageous for dry skin types. It may be too heavy for oily skin, potentially exacerbating oiliness or clogging pores, especially if used in excess. However, the linoleic acid component may help balance sebum production, so light application or diluted use might still benefit some individuals with oily skin.

Higher levels of palmitic acid in the cottonseed oil are the reason why it is one of the few oils stable in the beta crystal form, which is desirable in most solidified products because it promotes a smooth, workable consistency usually referred to as plasticity which is important in most formulations. At the same time, low levels of polyunsaturated fatty acids found in cottonseed oil result in a greater oxidative stability of cottonseed oil when compared to other high-linoleic oils (Pazzoti et al., 2018).

### 3.2 Changes in fatty acid composition associated with processing

Individual oil properties can also vary based on the extraction and refining methods used in its preparation. Both refined, winterized, and fully hydrogenated cottonseed oils offer distinct advantages in cosmetic and personal care formulations.

Refining alone does not significantly change the fatty acid profile and physiochemical properties of the cottonseed oil (Table 3). Both crude and refined cottonseed oils contain measurable amounts of diglycerides (5.8%) and minor monoglycerides (0.3%) that can be further fractionated (deMan et al., 1989). The diglyceride fatty acid composition is dominated by oleic acid (45%), linoleic acid (29%), and palmitic acid (23%) in a striking difference to the triglycerides (deMan et al., 1989). Full hydrogenation results in a spreadable cottonseed fat with melting point of 53 °C that is dominated by stearic (76%) and palmitic (22%) acids (Table 3).

**TABLE 3:** Changes in fatty acid profiles (%) during refining of cottonseed oil after (deMan et al., 1989; Dowd et al., 2010b; El-Mallah and El-Shami, 2011; Khan et al., 2021b; Ruzibayev et al., 2021).

	Olein (oil)						Stearin (palmitin)		
Cottonseed oil	Crude	Refined							
	(range)	Neutra-lized	Blea-ched	Deodo-rized	Winte-rized	Hydro-genated	Conven-tional	Solvent extracted	Hydro-genated
Myristic acid 14:0	0.6–1.4	0.8	0.8	0.8	trace	0.7	0.6	0.6	0.8
Palmitic acid 16:0	19.6–27.6	24.7	24.3	24.7	trace	22.3	37.3	52.1	38.3
Stearic acid 18:0	2.0–3.2	2.5	2.5	2.6	trace	76.2	4.8	2.1	56.7
Arachidic acid 20:0	0.2–0.5	0.4	0.4	0.5	trace	0.4	0.3	0.2	0.4
Palmitoleic 16:1 n-7	0.4–0.8	0.9	0.8	0.8	1.2	0	1.3	0.8	0
Oleic 18:1 n-9	12.8–22.2	17.3	17.6	18.1	25.4	trace	25.6	9.1	2.7
Linoleic 18:2 n-6	44.0–59.3	52.6	52.8	52.6	73.7	0	30.0	35.5	1.1
Linolenic 18:3 n-3	0.2–0.3	trace	trace	trace	0.2	0	0.1	0	0
Iodine value, g/100 g	103–111	109	110	110	114	4	96	72	5
Melting point, °C	0	−1	−1	−1	−12	53	24	35	59

Winterization of the refined cottonseed oil can be performed using either conventional or solvent-assisted chilling process that separates a solid stearin (palmitin) byproduct dominated by the palmitic acid (38%–52%) that can be further fully hydrogenated into a stearic acid (57%) and palmitic acid (38%) saturated cottonseed fat (Table 3). The high melting point makes it stable in solid form at room temperature, ideal for structuring personal care products in warm climates. The stearin also holds the potential to add thickness and a silky texture, thus replacing synthetic thickening agents like carbomers; work as a stabilizing base that improves firmness and melting properties, potentially replacing beeswax or paraffin; and add hardness to soaps without the drying effects of sodium stearate.

The resulting winterized cottonseed oil has a lower melting point (−12°C), allowing it to remain in a liquid state even in colder temperatures and a low to moderate viscosity, which contributes to its light, non-greasy texture, possibly replacing mineral oil in formulations. Its mild emollient properties make it effective for gentle cleansing, potentially substituting for sunflower or grapeseed oil.

### 3.3 Phytosterols

Cottonseed oil naturally contains a mixture of phytosterols (approx. 1%) dominated by β-sitosterol, campesterol, stigmasterol (also known as beta-stigmasterol or delta-5-stigmasterol), and isofucosterol (delta-5-avenasterol) (El-Mallah and El-Shami, 2011). This is 2–3 times higher than many other vegetable oils, and on par with the rice bran oil known to contain higher levels of plant phytosterols (Liu et al., 2023). Refining reduces the phytosterol content of cottonseed oil by about 15% (Table 4), and the remaining phytosterols can be recovered from the deodorizer distillate byproduct where they accumulate at the level of 10%–20% (El-Mallah and El-Shami, 2011). This has been successfully performed using molecular distillation (Jafri et al., 2014), and confirmed with the gas chromatography analysis (Verleyen et al., 2001).

**TABLE 4:** Phytosterol profiles during refining of cottonseed oil, summarized after (El-Mallah and El-Shami, 2011).

Phytosterols	Cottonseed oil			
mg/L (ppm)	Crude	Neutralized	Bleached	Deodorized
β-Sitosterol	8,199	7,713	7,495	6,869
Campesterol	874	855	800	725
Stigmasterol	255	240	224	210
Isofucosterol	171	162	161	144
Avenasterol	58	50	46	38

Under the normal physiological conditions, phytosterols are naturally secreted into the human skin surface lipids together with cholesterol at the rate of 3.2–8.5 mg/day for β-sitosterol and 0.2–0.4 mg/day for campesterol and stigmasterol (Bhattacharyya et al., 1972). In clinical settings, an ointment containing β-sitosterol in a base of beeswax and sesame oil improved management of skin burns, acute dermatitis, and post-operative wound healing in part due to its anti-inflammatory properties (Geara et al., 2018). The anti-inflammatory activity of β-sitosterol, campesterol, and stigmasterol was further confirmed in the activated keratinocyte and macrophages, as well as a preclinical model of psoriatic inflammation when applied topically at 1.4 mg/mL (Chang et al., 2023). Beta-sitosterol also showed moderate inhibition of 5α-reductase and induction of early hair follicle transition when delivered topically at the concentration of 2.4–3.6 mg/cm^2^ in a preclinical model of androgen-induced hair loss (Prabahar et al., 2022).

### 3.4 Tocopherols

Tocopherols are natural antioxidants that stop or delay primary oxidation, thus contributing to high oxidative stability of cottonseed oil and its formulations by scavenging lipid peroxyl radicals and protecting the cell membrane integrity (Salimath et al., 2021). Cottonseed oil contains high levels of tocopherols (approx. 0.1%), with γ-tocopherol (55%) and α-tocopherol (45%) in near equal concentrations, and trace amounts of the others. This is 1.5–2 times higher than many other vegetable oils, and on par with soybean oil, although high content of α-tocopherol was a unique feature that classified cottonseed and sunflower oil in the same category (Wen et al., 2020). Similar to sterols, refining of cottonseed oil reduces the tocopherol content of cottonseed oil by about 18% (Table 5). Cottonseed oil generally does not contain tocotrienols, although the biosynthesis pathway for tocotrienols can be successfully engineered into cotton plants (Salimath et al., 2021).

**TABLE 5:** Tocopherol profiles during refining of cottonseed oil, summarized after (El-Mallah and El-Shami, 2011).

Tocopherols	Cottonseed oil			
mg/L (ppm)	Crude	Neutralized	Bleached	Deodorized
γ-Tocopherol (7,8-dimethyl-tocopherol)	489	434	414	377
α-Tocopherol (5,7,8-trimethyl-tocopherol)	470	461	432	411
δ-Tocopherol (8-methyl-tocopherol)	6	5	3	2
β-Tocopherol (5,8-dimethyl-tocopherol)	4	2	1	0

While α-tocopherol is the predominant form of vitamin E in human tissues, other forms of vitamin E such as γ-tocopherol, δ-tocopherol and γ-tocotrienol and their natural carboxychromanol metabolites have stronger anti-oxidative and anti-inflammatory effects (Jiang, 2014). Under the normal physiological conditions, tocopherols are transported via the sebaceous glands and sebum, with the highest levels of vitamin E registered in sebum-rich facial skin (Ekanayake-Mudiyanselage and Thiele, 2006). Tocopherols are known in dermatology for their high UV radiation absorbing properties (Pedrelli et al., 2012). In a preclinical study, oral tocopherols also improved recovery of stitched skin wounds both in normal and diabetic states (Elsy et al., 2017). Synthetic α-tocopherol acetate increased the stratum corneum hydration when applied topically at 2.5% (Gehring et al., 1998), was substantially absorbed in skin, however no evidence of biotransformation within skin has been reported (Alberts et al., 1996). This can be associated with low incidence of α-tocopherol derivative-induced allergic contact dermatitis (Kosari et al., 2010), and switching to natural tocopherols found in vegetable oils may lead to better skin compatibility.

## 4 Current cosmetic applications of cottonseed oil

Cosmetic and personal care uses of vegetable oils are defined for external parts of the human body, teeth, or mucous membranes of the oral cavity to maintain them in good condition, change in appearance, protect, or correct body odors (EU 2009/1223) or cleansing, beautifying, promoting attractiveness, or altering the appearance (21 U.S.C. § 321(i)). In typical cosmetic formulations, the cottonseed oil is mostly used alone or in blends with the high-end botanical oils (argan, macademia, rosehip) in support for bulk applications where cost is a factor and refining is accepted. However, cottonseed oil also presents other opportunities in personal care as a prime ingredient, particularly for products focused on skin conditioning and moisturizing, thanks to its rich fatty acid profile, bioactive constituents, and potential for sustainable sourcing.

### 4.1 Carrier oils

The cottonseed oil is easily absorbed and has a moderately low comedogenic value (Table 2), thus it is often used as a carrier oil for other bioactive ingredients in cosmetic and skin care applications. This oil also contains a significant unsaponifiable fraction dominated by phytosterols and tocopherols that contribute to its greater oxidative stability when compared to other fixed oils (Taghvaei et al., 2014). The extended shelf life (Pazzoti et al., 2018), thermal stability, and absence of the inherent odor and color also make cottonseed oil a choice media in lipophilic botanical macerations. This provides both oxidative stability and a smooth, moisturizing quality without excessive oiliness.

### 4.2 Skin barrier function

Due to its high linoleic content, as well as a higher linoleic acid to oleic acid ratio, cottonseed oil may have a high skin barrier repair potential (Vaughn et al., 2018). Whether this effect is the same for healthy and damaged skin continues to be discussed (Moore et al., 2020; Poljšak and Kočevar Glavač, 2022).

Cottonseed oil also has a beta-type crystal structure similar to that of animal fats, which distinguishes it from many other vegetable oils (Ribeiro et al., 2014). This property makes cottonseed oil useful as a modifier to adjust the physical characteristics of other fats, such as cocoa butter (Ribeiro et al., 2013). This crystal habit is more similar to the structure of lipids naturally found in the skin, which allows cottonseed oil to blend effectively with the native lipid matrix and to enhance the skin barrier by reducing transepidermal water loss and preventing external irritants from penetrating.

Tolerance of the Johnson’s Natural baby lotion in a complex formulation with cottonseed oil and at least 95% naturally derived ingredients was evaluated on a torso, arms and legs of 1–36 months old children and showed no adverse effects in association with 37%–48% improvement in stratum corneum hydration (Coret et al., 2014). The cottonseed oil blends were also developed as a topical emollient lotion for improving the skin barrier and reversing microbial dysbiosis (Capone et al., 2022). Other potential uses may include recreation of a creamy and smooth texture in formulations, gentle cleansing and makeup removing, as well as massage slip and skin nourishment.

### 4.3 Hair follicles and sebaceous glands

Cottonseed oil contains significant amounts of palmitic acid, a natural precursor for production of human sebum components (Ge et al., 2003). Sebaceous glands are associated with hair follicles and lubricate both skin and hair to maintain hydration, a physical barrier, and a complex environment for the beneficial microbiome (Swaney et al., 2023). Cottonseed oil was used as a major ingredient (25%) in formulation of artificial sebum based on the chemical similarity to human sebum (Lu et al., 2009). This oil is a popular ingredient in some hair oils (Mannan et al., 2023), where it provides nourishing and conditioning benefits to the hair. High smoking point of cottonseed oil (230°C) makes it more suitable for heat styling.

### 4.4 UVB photoprotection

Although there are some limited reports indicating that the cottonseed oil blocks out about 20% of ultraviolet radiation (Korać et al., 2011), this translates to a low SPF value of 1-5, similar to other vegetable oils (Kaur and Saraf, 2010). This effect is most likely associated with a higher vitamin E (tocopherol) content of the cottonseed oil (Darr et al., 1996).

### 4.5 Cleaning soaps

Soaps formulated with different vegetable oils and natural herbal extracts are a major segment of the cosmetic and personal healthcare global market (Adigun et al., 2019). High linoleic and palmitic acid content of cottonseed oil defines the physiochemical soapmaking qualities of cottonseed oil (Table 2) and makes it suitable for manufacturing of soft (semi-liquid) soap bars and liquid soaps (Lynch, 1997). The cottonseed oil lacks a strong aroma, making it versatile for fragrance-free or scented soap products.

### 4.6 Biodegradable films

Biodegradable and edible (food grade) cosmetic formulations that reduce waste and can be consumed directly provide a novel experience and a sustainable alternative to promote environmental responsibility in cosmetics and personal care (Dini, 2024). Cottonseed oil can be successfully used to formulate biocompatible emulsion films that contain 1% glycerol and 3.4% lipids (Prus-Walendziak and Kozlowska, 2021). Since this oil is a byproduct of the cotton industry, it is manufactured at strict food-grade regulations and reduced raw materials costs, creating an economically attractive option for manufacturers focused on affordable food-safe cosmetics. For example, Lush’s biodegradable soap wrappers incorporate plant-derived waxes and oils to create dissolvable, plastic-free alternatives.

### 4.7 Delivery systems

The cottonseed oil is one of the solubilizing excipients often used in oral and injectable formulations. It can solubilize very lipophilic drugs and, following an intramuscular administration, serve as a sustained drug delivery depot for 2–4 weeks as the oils diffuses within the muscle tissue (Strickley, 2004). It also performs well in the microemulsion systems with improved solubility and penetration, outperforming oleic acid and Labrafil M1944 (Ryu et al., 2020). Cottonseed oil is compatible with a wide range of oils and ingredients, allowing it to blend well in complex formulations. For example, cottonseed oil-based oleogels with carnauba wax as a gelling agent have been successfully developed in consistencies from a thickened beverage to yogurt pudding in order to assist pediatric dosing and palliative care (Kirtane et al., 2022). Cottonseed oil showed good permeation enhancer abilities on par with sunflower oil (PATEL et al., 2018).

### 4.8 Fermentation substrates

Increasing number of conventional cosmetic ingredients change sources from synthetics to the fermentation-based products that support the sustainable growth of the industry (Pérez-Rivero and López-Gómez, 2023). Cottonseed oil was described as an advantageous fermentation substrate in production of natural sophorolipid biosurfactants that promote emulsification, wetting, solubilization, and detergency functions in cosmetic formulations (Qin et al., 2023).

### 4.9 Ethnopharmacological uses

The use of cottonseed oil to extract roots of pale bugloss (*Echium italicum* L.) and prepare an ointment to heal wounds and knife cuts was reported in Turkish herbal tradition (Simsek et al., 2004). Cottonseed oil and other plant parts were also used in various Ayurveda preparations to manage inflammation and wounds (Velmurugan et al., 2012). The cottonseed milk (paruthi paal) is popular in preparation of moisturizing skin and personal care products in the Tamil Nadu state of India (Kumar, 2019). Application of cottonseed oil was also reported to lighten skin spots and freckles (Raheman et al., 2012).

### 4.10 Other potential byproducts and their uses

Gossypol, a naturally present phenolic aldehyde in unrefined cottonseed oil, was shown to specifically inhibit lactic acid dehydrogenase (Judge et al., 2018). Lactic acid decreases skin extracellular pH and activates TGF-β1 signaling to promote the survival of skin myofibroblasts, overproduction of collagen, and fibrotic tissue degeneration. Thus, a possible management approach to skin fibrosis would be application of gossypol to suppress myofibroblasts differentiation and excessive collagen deposition in the skin tissue. Two other byproducts of cottonseed oil production, the hydrolyzed cottonseed protein (Essendoubi et al., 2022) and cottonseed oligosaccharides (Oberto et al., 2005) have been explored for heat protection of human hair fibers and reduction of hair chipping, respectively.

## 5 Regulatory and safety considerations

Cottonseed oil in cosmetics is listed under its INCI name as Gossypium Herbaceum (Cotton) Seed Oil. Regulatory checkpoints described for cottonseed oil are based on its agricultural background similar to other vegetable oils, levels of gossypol and cyclopropenoid acids that are eliminated during refining, and the need for strict quality control to ensure safety and efficacy in cosmetic formulations.

### 5.1 Raw materials

Cottonseed oil can be manufactured in several grades depending on its intended application. The pharmaceutical USP (US Pharmacopoeia) or NF (National Formulary) grade requires highest purity in excess of 99.9% with no binders, fillers, excipients, dyes, or unknown substances.

Food grade oil is the next highest grade as defined by the international standards for purity and classification established by the FCC (Food Chemical Codex) for cooking and ingestion by humans. Codex Alimentarius Commission (CAC) for fats and oils as a part of the FAO/WHO Food Standards Program also offers a set of internationally adopted standards that contribute to the safety and quality of food grade oils. Although not as frequent for cottonseed oil, adulterations and fraud of vegetable oils remains a significant issue (Green and Wang, 2023). Stricter quality controls, traceability measures, and advanced analytical techniques, such as HPLC and NMR are routinely used to detect adulteration and reinforce consumer confidence (Srivastava et al., 2024).

Cosmetic-grade oils are of lower quality, as the FDA (Food and Drug Administration) allows them to be only 70% pure, with the remaining oil blends and ingredients often undisclosed on the ingredient list. The organic cosmetic label still uses cosmetic grade ingredients. Lowest grade vegetable oils are of feed grade as regulated by the AAFCO (Association of American Feed Control Officials) or technical grade that do not meet the above criteria and should not be used in food and cosmetic applications.

### 5.2 Safety and sustainability

As a cosmetic ingredient, cottonseed oil was previously reviewed as safe (Cosmetic Ingredient Review Expert Panel, 2001; Raj et al., 2023). Routine tests for mycotoxins (aflatoxin), pesticide residues and heavy metals that can be introduced by the agricultural practices, environmental pollution, processing, and/or transportation are important (Xia et al., 2021). Due to a refining process, cottonseed oil is also generally non-irritating and hypoallergenic, suitable for sensitive skin, which is a major advantage over the cold-pressed or virgin oils that may contain proteins with IgE-binding epitopes, especially when derived from soybeans, wheat, peanuts, tree nuts, and sesame (Yu et al., 2016). Refined oils are also attractive for personal care because they have minimal aroma, reducing conflicts with other fragrances, pose low contamination risk, and generally offer a longer shelf life due to high oxidative stability.

Cottonseed oil is readily available and inexpensive, providing an eco-friendly alternative to more expensive oils. Its economic exploitation is closely tied to being a byproduct of cotton fiber production, making it a cost-effective source of vegetable oil (Prado et al., 2021). This dual-purpose utilization enhances sustainability by maximizing the value of cotton cultivation while reducing agricultural waste (Constable and Bange, 2015).

## 6 Conclusion

The use of oils in cosmetic and personal care products has a rich history rooted in natural sources, shaped by geographic and cultural influences. In recent years, consumer demand for eco-friendly, sustainable options has revitalized interest in plant-based oils, including underutilized options like cottonseed oil. Distinctive fatty acid profile of cottonseed oil, with its beneficial linoleic to oleic acid ratio and a higher-than-expected level of palmitic acid, contributes to its unique characteristics in terms of stability, texture, and the way it interacts with other lipids. As the cosmetic industry shifts toward more naturally derived ingredients, cottonseed oil holds potential as a versatile, affordable, and safe component suitable for diverse personal care applications. Further research is needed to assess its long-term efficacy, stability in complex formulations, suitability for sensitive skin, and its interaction with other bioactive ingredients.
